# Synthesis of Polypyrrole/Reduced Graphene Oxide Hybrids via Hydrothermal Treatment for Energy Storage Applications

**DOI:** 10.3390/ma13102273

**Published:** 2020-05-15

**Authors:** Adam Moyseowicz, Krzysztof Pająk, Katarzyna Gajewska, Grażyna Gryglewicz

**Affiliations:** Department of Process Engineering and Technology of Polymer and Carbon Materials, Faculty of Chemistry, Wrocław University of Science and Technology, Gdańska 7/9, 50-344 Wrocław, Poland; adam.moyseowicz@pwr.edu.pl (A.M.); 204817@student.pwr.edu.pl (K.P.); 217791@student.pwr.edu.pl (K.G.)

**Keywords:** self-assembly method, hydrothermal synthesis, polypyrrole-graphene composites, supercapacitor

## Abstract

Herein, we propose hydrothermal treatment as a facile and environmentally friendly approach for the synthesis of polypyrrole/reduced graphene oxide hybrids. A series of self-assembled hybrid materials with different component mass ratios of conductive polymer to graphene oxide was prepared. The morphology, porous structure, chemical composition and electrochemical performance of the synthesized hybrids as electrode materials for supercapacitors were investigated. Nitrogen sorption analysis at 77 K revealed significant changes in the textural development of the synthesized materials, presenting specific surface areas ranging from 25 to 199 m^2^ g^−1^. The combination of the pseudocapacitive polypyrrole and robust graphene material resulted in hybrids with excellent electrochemical properties, which achieved specific capacitances as high as 198 F g^−1^ at a current density of 20 A g^−1^ and retained up to 92% of their initial capacitance after 3000 charge–discharge cycles. We found that a suitable morphology and chemical composition are key factors that determine the electrochemical properties of polypyrrole/reduced graphene oxide hybrid materials.

## 1. Introduction

The ever-growing demand for efficient and durable energy storage devices warrants the development of novel materials and solutions that will meet our expectations. Supercapacitors are effective for this purpose, as they are characterized by high power, good energy densities, excellent long-term performance and a wide range of possible applications in different conditions [1]. The core factors that determine the electrochemical performance of the supercapacitors are electrodes, thus an appropriate design and fabrication of the electrodes with suitable structural and chemical properties are crucial to develop high-performance energy storage devices [2]. The charge storage mechanism in supercapacitors is strictly dependent on the type of electrode material used and can be classified into two categories: electrical double layer (EDL) capacitance and pseudocapacitance [3]. The former takes advantage of the electrostatic charge accumulation at the surface of highly porous carbon materials, including activated carbons [2], carbon nanotubes/nanofibers [4] and graphene-related materials [5]. The latter energy storage mechanism is based on Faradaic redox reactions of heteroatom-doped carbons [6], transition metal compounds (oxides [7], sulfides [8] or nitrides [9]) and conductive polymers [10,11].

In order to improve the electrochemical characteristics of electrodes, hybrids or composites were proposed, which combine carbon-based materials with pseudocapacitive materials and show the most promising results due to the synergistic effects of the used components [12,13]. Conductive polymer-graphene hybrids are a unique group of flexible and often binder-free materials with exceptional electrochemical performance as supercapacitor electrodes or sensing platforms [11,14,15]. Among the most promising conductive polymers, researchers focus on polyaniline (PANI), polypyrrole (PPy), polythiophene (PTh) and their derivatives [11]. PPy is characterized by high conductivity, good redox reversibility and environmental friendliness; however, due to swelling and polymer chain degradation, it exhibits insufficient cyclic stability [16]. Therefore, reduced graphene oxide (rGO) as an additional component for hybrid materials offers a robust scaffold for the polymer matrix, excellent conductivity and electrochemical stability [14]. Previously reported research involving different types of polypyrrole–graphene hybrids indicated their great potential as electrodes for supercapacitor applications [17,18,19,20,21]; however, obtaining high capacitance values along with very good long-term performance is still a challenge to be met. 

Hydrothermal treatment is an attractive and environmentally friendly approach for the preparation of three-dimensional (3D) nanostructures, which yields materials with developed porosity, interconnected networks and short electron transportation paths [22]. However, hydrothermal synthesis is rarely used for preparation of the conductive polymer/graphene-based composites, which suffer from a low specific surface area, resulting in an inhibited charge transfer at the electrode/electrolyte interface [23,24].

This work presents a facile, two-step synthesis of polypyrrole/reduced graphene oxide (PPy/GO-HT) hybrids, which involves a chemical polymerization of pyrrole, followed by a hydrothermal self-assembly of PPy and a graphene oxide (GO) dispersion. We performed a systematic study on the influence of the component mass ratio on the composite porous structure, morphology and chemical characteristics. PPy/GO-HT hybrids with specified physical and chemical properties, used as electrode materials for supercapacitors operating in an aqueous electrolyte, exhibited high specific capacitance values and a remarkable cyclic stability. The obtained results indicate that the chemical composition of the PPy/GO-HT hybrids is one of the most crucial factors influencing composite electrochemical performance. The hydrothermal approach offers extensive possibilities for synthesis in order to obtain hybrids with suitable physical and chemical characteristics. Additionally, the electrodes prepared from PPy/GO-HT hybrids did not require an additional binder, facilitating the fabrication process, which further emphasizes their potential application in flexible or asymmetric supercapacitors. 

## 2. Materials and Methods 

### 2.1. Preparation of Graphene Oxide

Synthetic graphite supplied by Sigma-Aldrich (Poznan, Poland) was used for GO preparation using a modified Hummers method [25]. The procedure was previously reported in our work [26], and detailed procedure parameters are included in Electronic Supplementary Information.

### 2.2. Preparation of Polypyrrole

The reagents used for the synthesis of polypyrrole (pyrrole, hydrochloric acid, ammonium persulfate and Triton X-100^®^) were of analytical grade and supplied by Sigma-Aldrich, Poznan, Poland. Prior to synthesis, the pyrrole monomer was stored under an inert atmosphere to prevent oxidation. The polymerization procedure was as follows: 1 g of a pyrrole monomer was dispersed with 10 mg of Triton X-100^®^ in 40 mL of Milli-Q water and 20 mL of 1 M HCl for an hour. Then, an adequate amount of ammonium persulfate (APS), as a polymerization initiator, was added, with a molar ratio of pyrrole to APS of 1:1. The polymerization mixture was held at temperature of 0–5 °C for 6 h without mixing, and the resultant material was denoted as PPy. Finally, the obtained polypyrrole was filtered and washed with Milli-Q water and methanol to a neutral pH and dried at 60 °C under vacuum for 12 h.

### 2.3. Hydrothermal Self-Assembly of PPy/GO-HT Hybrids

The polypyrrole/reduced graphene oxide hybrids were synthesized via a one-step, self-assembly hydrothermal treatment with different mass ratios of GO to PPy. The resultant samples were denoted as PPy/GO-HT-X:Y, where X and Y refer to the initial mass contributions of polypyrrole and graphene oxide, respectively. Briefly, for the synthesis of PPy/GO-HT-3:1, 100 mL of GO solution (1.5 mg mL^−1^) and 450 mg of PPy powder were mixed together and ultrasonicated for 10 min. Next, the as-received suspension was transferred to the stainless-steel autoclave in order to reduce the GO under hydrothermal conditions at 180 °C for 5 h without mixture agitation. After the reaction, the autoclave was cooled to room temperature. The resultant deposit was washed three times with distilled water to a neutral pH (~7) and then dried under vacuum at 60 °C for 12 h. For the rest of the hybrids, the PPy and GO masses in the initial mixture were, respectively, 150 and 150 mg for PPy/GO-HT-1:1, 50 and 150 mg for PPy/GO-HT-1:3, and 16.6 and 150 mg for PPy/GO-HT-1:9. For comparison, rGO was prepared using the same self-assembly hydrothermal conditions without the PPy.

### 2.4. Characterization Methods

The as-prepared materials were characterized using field emission scanning electron microscopy (FESEM, MERLIN Zeiss, Jena, Germany) and X-ray photoelectron spectroscopy (XPS, PHI 5000 VersaProbe, Chigasaki, Japan). Sample charging was corrected using the C1s peak at a binding energy of 284.6 eV as an internal standard, and curve fittings were performed using the CasaXPS software (version 2.3.17, Casa Software Ltd, Teignmouth, UK). The porous texture of the materials was analyzed using N_2_ sorption at 77 K (Autosorb IQ gas sorption analyzer, Quantachrome). The specific surface area (S_BET_) was calculated using the Brunauer–Emmett–Teller (BET) method based on the adsorption data in the relative pressure (p/p_0_) range of 0.02–0.2. The total pore volume (V_total_) was determined at the relative pressure of p/p_0_ = 0.96. The Dubinin–Radushkevich equation was applied to estimate the micropore volume (V_mic_). The mesopore volume (V_mes_) was calculated as the difference between V_total_ and V_mic_. Pore size distributions were determined using the quenched solid density functional theory (QSDFT).

### 2.5. Electrochemical Measurements

Investigations of the electrodes’ electrochemical performance were conducted in a three-electrode setup (Swagelok™, Bron, France) in a 1 M H_2_SO_4_ electrolyte using BioLogic VSP potentiostat-galvanostat. The electrochemical performance of the materials was investigated using cyclic voltammetry (CV) measurements and galvanostatic charge-discharge (GCD) experiments at different current densities and electrochemical impedance spectroscopy (EIS). The detailed procedure for the electrochemical characterization is included in Electronic Supplementary Information. 

## 3. Results and Discussion

### 3.1. Porous Texture of the Hybrid Materials and Their Precursors

**Figure 1:**
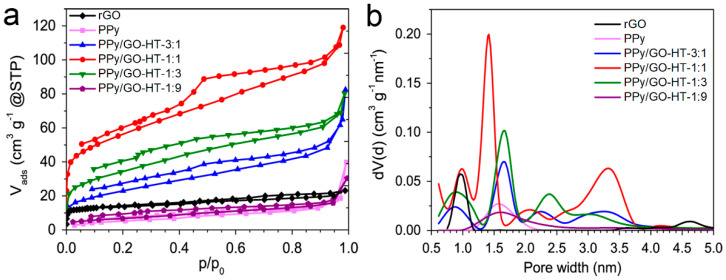
(**a**) N_2_ sorption isotherms; (**b**) Quenched solid density functional theory (QSDFT) pore size distribution of reduced graphene oxide (rGO), polypyrrole (PPy) and polypyrrole/reduced graphene oxide (PPy/GO-HT) hybrids.

The nitrogen adsorption–desorption at 77 K and the QSDFT pore size distribution profiles of the PPy/GO-HT hybrids and their precursors are presented in Figure 1. The textural parameters calculated from the N_2_ adsorption data are included in Table 1. Distinct differences in the isotherms between rGO and the polymer-based materials indicate their divergent characteristics (Figure 1a). According to the IUPAC classification, rGO exhibits a type I isotherm associated with microporous materials, while PPy and the PPy/GO-HT-1:9 composite present a type III isotherm, indicating poorly developed porosity. However, hybrids with 3:1, 1:1 and 1:3 PPy to GO mass ratios exhibit a type IV isotherm with an H2 hysteresis loop [27]. Hybrid PPy/GO-HT materials present mixed microporous and mesoporous traits with clearly visible maxima on the PSD profiles at different values between 1 and 3.5 nm (Figure 1b). Graphene material shows one strong maximum at a pore width of 1 nm. The specific surface areas (S_BET_) of PPy and rGO are 20 and 51 m^2^ g^−1^, respectively (Table 1). The relatively low porosity of the rGO is probably related to the significant restacking of the graphene nanosheets, which occurs under hydrothermal conditions without mixing due to the van der Waals interactions [28,29]. However, the combination of 25 to 75 wt.% of PPy with GO dispersion in a hydrothermal-assisted, self-assembled synthesis resulted in binary hybrids with a more developed porous structure compared with their precursors. The highest development of binary material porosity was when an equal mass ratio of PPy to GO (1:1) was applied, achieving a S_BET_ area of 199 m^2^ g^−1^ (Table 1). Such a phenomenon can be explained by the self-organization of PPy aggregates on the GO surface, which act as spacing agents during hydrothermal synthesis and prevent the significant restacking of the graphene nanosheets [28,30]. Additionally, the introduction of the polymer nanoparticles to the GO dispersion led to the development of mesopores, increasing their contribution from 32% for rGO to 54–67% for PPy/GO-HT hybrids (Table 1). In the case of PPy/GO-HT-1:9, the minor addition of a non-porous PPy resulted in a decrease in the composite S_BET_ area due to the blockage of the rGO pores with conductive polymer [16].

### 3.2. Morphology and Chemical Structure of the Binary Composites and Their Precursors

**Figure 2:**
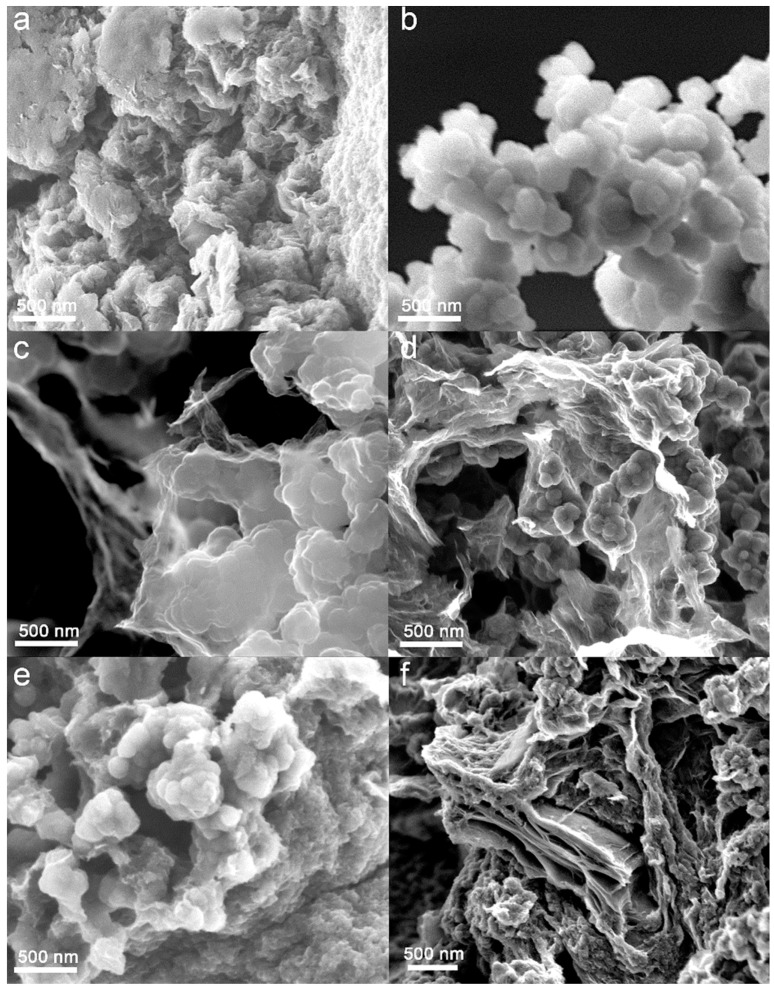
Field emission scanning electron microscopy (FESEM) images of (**a**) rGO, (**b**) PPy, (**c**) PPy/GO-HT-3:1, (**d**) PPy/GO-HT-1:1, (**e**) PPy/GO-HT-1:3 and (**f**) PPy/GO-HT-1:9.

The morphologies of the PPy/GO-HT hybrids and their precursors are shown in Figure 2. The rGO is composed of strongly packed and distorted nanosheets (Figure 2a), usually observed for the graphene materials obtained from hydrothermal synthesis [6,26]. PPy shows a typical aggregated structure of nanospherical particles with a diameter up to 200 nm, similar to a cauliflower (Figure 2b). Furthermore, significant differences in morphology between the PPy/GO-HT hybrids with various mass ratios of the components were observed. In PPy/GO-HT-3:1, thin rGO layers of 1.5 µm wide only partially cover the polymer aggregates due to the PPy high initial load, which can result in a short circuit (Figure 2c) [17]. Due to the balanced amounts of both precursors and electrostatic π–π interactions in the PPy/GO-HT-1:1 hybrid, PPy nanoparticles, usually smaller than 100 nm, are interweaved within graphene nanosheets (Figure 2d), resulting in an inhibition of GO restacking under hydrothermal conditions [23,31]. In the case of PPy/GO-HT-1:3, a separation between the rGO and PPy phases is observed, which may suggest that the polymeric component is mainly present at the surface of the material (Figure 2e). Moreover, when GO contributes to 90% of the initial synthesis mixture, the resultant hybrid material presents an interconnected graphene structure with small PPy nanospheres (<75 nm) on the composite surface (Figure 2f). At a high GO mass load, a significant restacking of the graphene layers occurred, which explains the decrease in the specific surface area of PPy/GO-HT-1:9 in comparison with other hybrid materials. The FESEM observations clearly demonstrate that the component mass ratio determines the final structure of the PPy/GO-HT composites synthesized under hydrothermal conditions without mixing.

**Figure 3:**
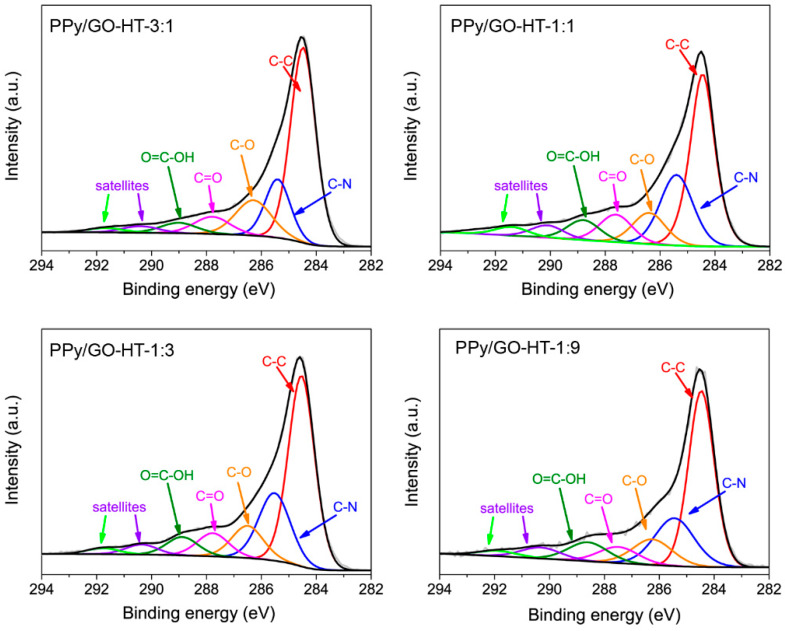
C1s high-resolution spectra of the PPy/GO-HT hybrids with different component mass ratios.

The results obtained from the XPS analysis for the hybrid materials and their precursors are presented in Table 2. The rGO is characterized by a high amount of oxygen (17.1 at.%). The C1s high-resolution deconvoluted spectrum shows a significant contribution of hydroxyl, carbonyl and carboxyl groups, as well as of carbon in sp^3^ hybridization (Figure S1). These results indicate only the partial reduction of GO during the hydrothermal synthesis [23,26]. The C1s core level spectra for PPy and the PPy/GO-HT hybrids were deconvoluted into seven components: C–C (284.5), C–N (285.6 eV), C–O (286.1 eV), carbonyl/quinone (287.5 eV), carboxyl groups (288.6 eV) and two satellite peaks for the carbon–carbon and carbon–nitrogen bonds at 290 and 291.6 eV, respectively (Figure 3 and Figure S1) [16,18,32]. Slight differences in the contribution of oxygen functionalities between the PPy/GO-HT hybrids are observed due to the various amounts of PPy introduced into the GO dispersion. As the synthesis procedure is based on a self-assembling process, we assume that different kinetics of the GO reduction occur, suggesting competition between PPy and GO interactions and GO reduction [33]. It should be noted that oxygen functionalities originate not only from graphene material but also from oxalate groups formed during the polymerization process with APS as an initiator [34]. 

**Figure 4:**
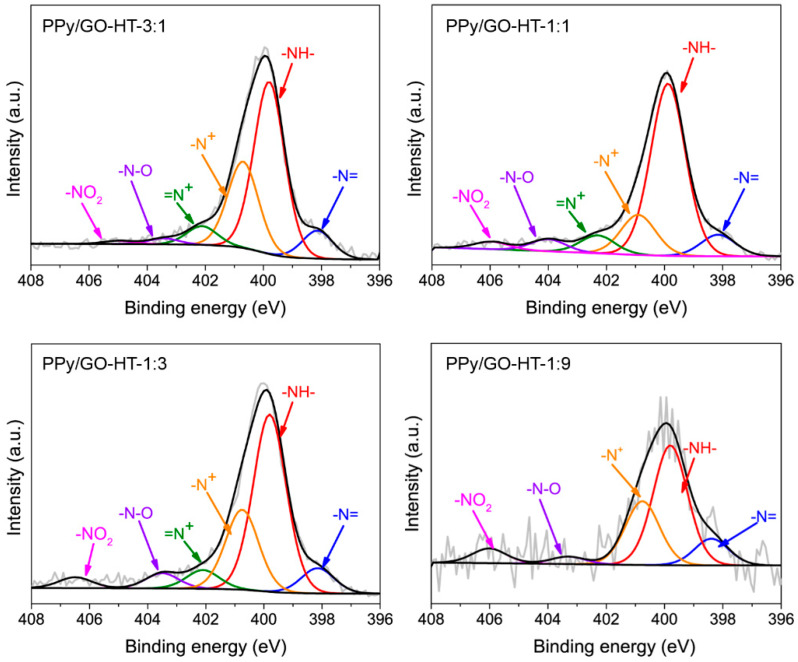
N1s high-resolution spectra of the PPy/GO-HT hybrids with different component mass ratios.

The amount of nitrogen decreases as the mass contribution of GO increases; however, a significant amount of PPy is still observed at the surface of the composites. The nitrogen content decreases from 12.2 at.% for PPy to 4.1 at.% for the hybrid materials with 90% GO in the initial synthesis mixture (Table 2). The deconvoluted N1s core level spectra for PPy and the PPy/GO-HT hybrids present four peaks corresponding to imine –N= (398.1 eV), amine –NH– (399.9 eV), positively charged nitrogen species of amine (400.8 eV) and imine (402.2 eV) groups (Figure 4 and Figure S2). Additionally, the PPy/GO-HT hybrids exhibit two extra peaks around 403.5 eV and 406 eV attributed to the oxygen-chemisorbed nitrogen and -NO_2_ groups, respectively [15,32,34,35]. The most abundant nitrogen species in the PPy/GO-HT hybrids are amine groups (~54%–63%), which originate from the five-membered pyrrolic rings (Table 2). The doping level expressed as a ratio of positively charged nitrogen species to the total nitrogen content is between 22% and 33% for the PPy/GO-HT hybrids and their polymeric precursor. Surprisingly, strong oxidation of the PPy occurs during hydrothermal synthesis, resulting in a partial conversion of amine to imine nitrogen, whose contribution increases up to four times, from 3% to 12%. Moreover, the hydrothermal self-assembly method for PPy/GO-HT hybrids led to the formation of nitro groups and oxygen-chemisorbed nitrogen. The results suggest that a highly oxidized graphene precursor undergoes reduction with polypyrrole amine groups [36].

### 3.3. Electrochemical Performance of the PPy/GO-HT Hybrids

**Figure 5:**
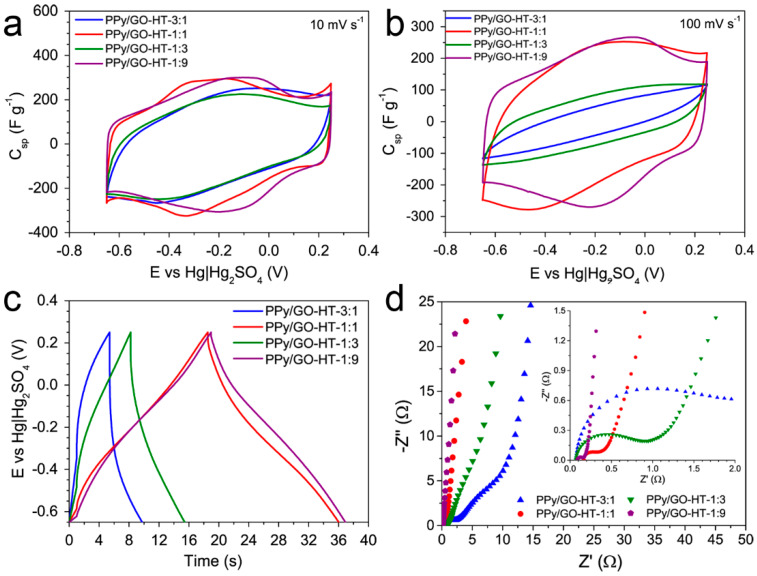
Electrochemical performance of PPy/GO-HT hybrids. Cyclic voltammetry (CV) curves at scan rates of (**a**) 10 mV s^−1^ and (**b**) 100 mV s^−1^; (**c**) galvanostatic charge–discharge profiles at a current density of 10 A g^−1^; (**d**) Nyquist plots with an inset representing the high-frequency range.

The electrochemical properties of the as-prepared materials were measured in a three-electrode system. The CV curves of the PPy/GO-HT hybrids at scan rates of 10 and 100 mV s^−1^ are shown in Figure 5a,b, respectively. All the composites exhibit a quasi-rectangular shape with redox humps on the CV curve, indicating their pseudocapacitive properties, which originate from the applied precursors. The CV curve shapes of PPy/GO-HT-1:1 and PPy/GO-HT-1:9 resemble that of the rGO, while those for PPy/GO-HT-3:1 and PPy/GO-HT-1:3 are similar to that for the PPy (Figure S3). However, the contribution of the EDL capacitance to the overall electrochemical performance is negligible, as the specific surface area of the hybrids does not exceed 200 m^2^ g^−1^. The pseudocapacitive humps observed in the CV profile of rGO are related to the high amount of oxygen functionalities, which contribute to the overall capacitance, especially in an acidic electrolyte [37]. Furthermore, the shape of the CV curves is changed drastically when the scan rate is increased up to 100 mV s^−1^ (Figure 5b). The significant deterioration of capacitive properties of the binary hybrids with 3:1 and 1:3 GO to PPy mass ratios, as well as the PPy itself (Figure S3b), was observed, indicating limited charge transfer within the electrode material [38]. At the high scan rate of 100 mV s^−1^, PPy/GO-HT-1:1 and PPy/GO-HT-1:9 show the best electrochemical performance. The former composite is characterized by the most developed porous structure and a high conductive polymer contribution, while the latter hybrid displays excellent charge propagation, which can be attributed to the interconnected and highly conductive graphene structure [19,39].

Figure 5c presents the GCD profiles of the PPy/GO-HT hybrids at a current density of 10 A g^−1^. The synthesized materials are characterized by a quasi-symmetric triangular shape of the charge–discharge curves, indicating a capacitive behavior [20,40]. At a high current density of 10 A g^−1^, when charge transfer occurs rapidly at the electrode/electrolyte interface, the composites with 3:1 and 1:3 PPy to GO mass ratios present much worse capacitive performance compared with the 1:1 and 1:9 hybrids, which confirms the results from the CV measurements. A comparable electrochemical performance of PPy/GO-HT-1:1 and PPy/GO-HT-1:9 was observed, although they differ significantly in terms of porous texture and morphology.

The results of the EIS measurements in the form of the Nyquist plots are presented in Figure 5d for the PPy/GO-HT hybrids and in Figure S4 for their precursors. The solution resistance (R_s_) determined from the intercept of the Z′ axis for all materials is in the range of 0.07–0.17 Ω [41,42]. However, significant differences between the binary materials are observed in the high frequency region of the Nyquist plot. PPy/GO-HT-3:1 and PPy/GO-HT-1:3 show larger semicircles, the diameters of which—1.5 and 0.8 Ω, respectively—are proportional to the charge transfer resistance (R_ct_), indicating distorted conductivity and limited ionic transfer within the electrode material [38,43,44]. Additionally, the semicircle attributed to the non-EDL charge storage kinetics of PPy/GO-HT-3:1 originates from the high contribution of the composite pseudocapacitive PPy component. The decreased conductivity of PPy/GO-HT-1:3 is due to the separated phases of the components, thus graphene nanosheets do not provide efficient charge transport within electrode material. Furthermore, the highly developed porous structure of PPy/GO-HT-1:1 and very high load of the graphene material in the PPy/GO-HT-1:9 composite resulted in fast charge transfer kinetics, presenting R_ct_ of 0.2 and 0.07 Ω, respectively, the latter of which is close to that of the rGO itself (0.06 Ω). A straight vertical line in the low frequency range reflects the capacitive performance of the system, and the more the line is inclined towards an imaginary part of the impedance, the more it corresponds to the characteristics of an ideal capacitor [23,45].

**Figure 6:**
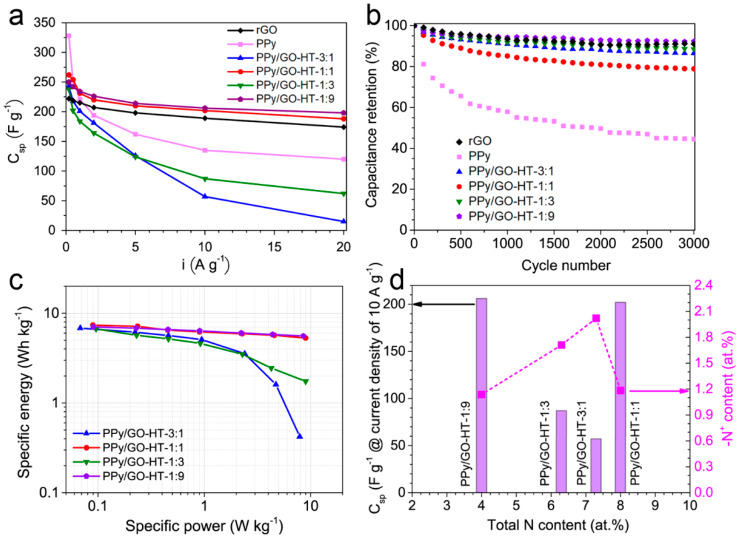
(**a**) Specific capacitance versus discharge current density for rGO, PPy and the PPy/GO-HT hybrids; (**b**) Electrode cycling stability at a current density of 2 A g^−^^1^ for the PPy/GO-HT hybrids and their precursors; (**c**) Ragone plot of the specific energy density versus the specific power density of the PPy/GO-HT hybrids; (**d**) Relationship between the chemical composition and specific capacitance of the hybrid materials with different component mass ratios at a current density of 10 A g^−1^.

The specific capacitance values at a current density of 0.2 A g^−1^ are found to be 328, 243, 262, 238, 250 and 220 F g^−1^ for PPy, PPy/GO-HT-3:1, PPy/GO-HT-1:1, PPy/GO-HT-1:3, PPy/GO-HT-1:9 and rGO, respectively (Figure 6a). The highest rate capability among all materials was observed for the PPy/GO-HT-1:9 hybrid with the lowest internal resistance, which maintained 79% of the specific capacitance with an increase in the current density from 0.2 to 20 A g^−1^ (198 F g^−1^). The capacitive performance of the PPy/GO-HT-1:9 hybrid at very high current regimes is superior to that of many other reported PPy-based composites as electrode materials working in aqueous electrolytes (Table S1). The significant deterioration of the electrochemical performance at high current regimes of the binary materials with 3:1 and 1:3 PPy to GO mass ratios indicates a failure to efficiently utilize the Faradaic redox reactions of PPy.

The long-term performance of the electrode material is an important factor in the evaluation of its applicability in energy storage devices, and the results are presented in Figure 6b. PPy exhibited a capacitance retention of 45% after 3000 charge–discharge cycles at a current density of 2 A g^−1^ due to the repeated swelling and shrinkage of the electrode material, which induced fast polymer structure decay [10,31]. For comparison, rGO showed an excellent capacitance retention of 91% after the cyclic stability test. The obtained results clearly show that the implementation of the robust graphene material with the conductive polymer greatly enhances the stability of the electrode material. The PPy/GO-HT hybrids maintained between 79% and 92% of their initial capacitance after 3000 charge–discharge cycles. The slightly lower stability of PPy/GO-HT-1:1 compared to the other composites could be related to the facilitated penetration of electrolyte ions into the electrode porous structure (Figure 6b). The PPy/GO-HT hybrids present very good long-term performance compared to other previously reported PPy and graphene-based materials in an aqueous electrolyte (Table S1) [21,31,42,46,47].

Figure 6c shows the Ragone plot corresponding to the energy–power relationship for the symmetrical device based on the PPy/GO-HT composite electrodes [48,49]. The highest specific energy density of 7.4 Wh kg^−1^ at a power density of 0.09 kW kg^−1^ was observed for the PPy/GO-HT-1:1 hybrid, which is comparable with that for commercial supercapacitors working in organic electrolytes [50]. Additionally, remarkable rate capability of the PPy/GO-HT-1:9 resulted in a specific energy density of 5.6 Wh kg^−1^ at a high power density of 8.5 kW kg^−1^. 

Electrochemical measurements in an aqueous acidic electrolyte showed interesting results for two hybrid materials that differ significantly in terms of morphology and porous structure; however, their electrochemical performance is similar. We revealed that chemical composition is a crucial factor that influences the capacitive characteristics of the hybrid polypyrrole-reduced graphene oxide materials, especially at high current densities. Figure 6d presents the correlation between the composite specific capacitance at a current density of 10 A g^−1^ and the total nitrogen content and calculated amount of protonated amine -N^+^ (polaron). For the binary materials with PPy to GO mass ratios of 3:1, 1:1 and 1:3, the total amount of nitrogen is similar, ranging from 6.3 to 8 at.%, while PPy/GO-HT-1:9 was characterized by an almost two-fold lower amount of nitrogen compared to the composite with a 1:1 mass ratio (Table 2). Although the PPy/GO-HT hybrids exhibit comparable doping levels of N^+^/N in the range of 22–33%, the differences in their electrochemical performance can be related to the content of polaron nitrogen present in the electrode material. PPy/GO-HT-1:1 and PPy/GO-HT-1:9 show 1.2 and 1.1 at.% of –N^+^ species, respectively, while much higher values are obtained for PPy/GO-HT-3:1 and PPy/GO-HT-1:3 (2.0 and 1.7 at.%, respectively). These findings suggest that the optimal amount of nitrogen polaron species results in a remarkable electrochemical performance for PPy/rGO hybrids [21,51]. The high doping levels of the polymer component in PPy/GO-HT-3:1 and PPy/GO-HT-1:3 suggest that during a hydrothermal self-assembly process, a migration of the $SO_{4}^{2-}$ anions occurs, accompanied by interactions with polypyrrole nitrogen. These mechanisms can lead to the quaternization of the nitrogen, thus resulting in decreased stability and increased resistivity [52].

## 4. Conclusions

In summary, a series of PPy/GO-HT hybrids with excellent electrochemical performance were fabricated via a hydrothermal self-assembly process. An additional advantage of the binary composites was the removal of the conventional binders by applying a conductive polymer. By varying the mass ratios of the initial components (PPy and GO), binary materials with different porosities, morphologies and chemical compositions were obtained. Despite the significant differences in morphology and porous structure, the composites with initial mass ratios of 1:1 and 1:9 showed comparable electrochemical characteristics due to the similarities in chemical composition and amount of protonated amine nitrogen. At the low current density of 0.2 A g^−1^, the highest specific capacitance of 262 F g^−1^ was recorded for PPy/GO-HT-1:1, while at the high current density of 20 A g^−1^, the PPy/GO-HT-1:9 hybrid exhibited the best rate capability and capacitive performance of 198 F g^-1^. The high load of interconnected graphene material in PPy/GO-HT-1:9 resulted in remarkable conductivity and stability under cycling; it retained 92% of its initial capacitance after 3000 charge–discharge cycles at a current load of 2 A g^−1^. Finally, a specific energy density of 7.4 Wh kg^−^^1^ at a power density of 0.09 kW kg^−1^ was achieved by PPy/GO-HT-1:1. This study shows the great potential of hydrothermal treatment for the synthesis of conductive polymer/graphene-based hybrids as high-performance electrode materials for supercapacitors. 

## Figures and Tables

**Table 1:** Textural parameters of rGO, PPy and the PPy/GO-HT hybrids obtained from the N_2_ sorption analysis.

Material	S_BET_	V_total_	V_mes_	V_mes_/V_total_
	(m^2^ g^−1^)	(cm^3^ g^−1^)	(cm^3^ g^−1^)	
rGO	51	0.031	0.010	0.32
PPy	20	0.024	0.022	0.76
PPy/GO-HT-3:1	82	0.095	0.064	0.67
PPy/GO-HT-1:1	199	0.168	0.091	0.54
PPy/GO-HT-1:3	122	0.105	0.060	0.57
PPy/GO-HT-1:9	25	0.025	0.016	0.64

**Table 2:** Chemical composition of the rGO, PPy and PPy/GO-HT hybrids as determined by XPS.

Material	C	N	O	–N=	–NH–	Doping Level N^+^/N
	at.%			%		
rGO	82.9	–	17.1	–	**–**	–
PPy	75	12.2	12.8	3	70	27
PPy/GO-HT-3:1	81.5	7.3	11.2	9	55	33
PPy/GO-HT-1:1	78.2	8	13.8	8	63	22
PPy/GO-HT-1:3	80.3	6.3	13.4	8	55	31
PPy/GO-HT-1:9	82	4.1	13.9	12	57	28

## Supplementary Materials

The following are available online at https://www.mdpi.com/1996-1944/13/10/2273/s1, Graphene oxide preparation, Electrochemical measurements details, Figure S1. C1s high-resolution spectra of reduced graphene oxide (rGO) and polypyrrole (PPy); Figure S2. N1s high-resolution spectra of PPy; Figure S3. Cyclic voltammetry (CV) curves for (**a**) rGO and (**b**) PPy at the various scan rates in a 1 M H_2_SO_4_ electrolyte and Figure S4. Nyquist plots of rGO and PPy with an inset representing the high-frequency range, Comparison of the PPy and carbon nanostructure composites electrochemical performance in aqueous electrolytes, Table S1. Comparison of the PPy and carbon nanostructure composites’ electrochemical performance in aqueous electrolytes.
