# Supplementary material for: Synthesis of Polypyrrole/Reduced Graphene Oxide Hybrids via Hydrothermal Treatment for Energy Storage Applications
Source: Materials (Basel). 2020 May 15;13(10):2273. doi: 10.3390/ma13102273 (PMC7287821; doi:10.3390/ma13102273)
Supplement: Supplementary file 1 [file materials-13-02273-s001.pdf]

materials

Article

Synthesis of Polypyrrole/Reduced Graphene Oxide Hybrids via Hydrothermal Treatment for Energy Storage Applications

Adam Moyseowicz, Krzysztof Pająk, Katarzyna Gajewska and Grażyna Gryglewicz *

Department of Process Engineering and Technology of Polymer and Carbon Materials, Faculty of Chemistry, Wrocław University of Science and Technology, Gdańska 7/9, 50-344 Wrocław, Poland; adam.moyseowicz@pwr.edu.pl (A.M.); 204817@student.pwr.edu.pl (K.P.); 217791@student.pwr.edu.pl (K.G.)
* Correspondence: grazyna.gryglewicz@pwr.edu.pl; Tel.: +48-71-320-6398; fax: +48-71-320-6506.

Received: 20 April 2020; Accepted: 12 May 2020; Published: date

Preparation of Graphene Oxide

Briefly, 1 g of graphite was mixed with $NaNO_3$ and concentrated H_2SO_4, followed by the addition of $KMnO_4$ to the mixture under steady stirring, in which the temperature was maintained at 35 °C for 3 h. Afterwards, an aqueous solution of H_2O_2 was introduced into the flask. The as-received product was washed with ultrapure Milli-Q water and centrifuged. Then, the graphite oxide was treated with diluted HCl and washed with water until a neutral pH was reached. An aqueous dispersion of GO (1.2 mg mL^{-1}) was prepared via ultrasonication for 6 h.

Electrochemical Measurements

The electrochemical performance of the materials was first investigated via cyclic voltammetry measurements (CV) at a scan rate of 1–100 mV s^{-1} in the potential range of −0.65 to 0.25 V. Moreover, galvanostatic charge–discharge (GCD) experiments at current densities ranging from 0.2 to 20 A g^{-1} and electrochemical impedance spectroscopy (EIS) measurements under an open circuit potential in the frequency range of 100 kHz to 10 mHz were performed. The reference and counter electrodes were $Hg|Hg_2SO_4$ and pitch-based KOH-activated carbon, respectively. The PPy and PPy/GO electrodes were binder-free, containing 95 wt.% of the active material and 5 wt.% of carbon black (CB). The electrodes from rGO contained 90 wt.% of the active material, 5 wt.% of polyvinylidene fluoride (PVDF, Kynar Flex) and 5 wt.% of CB.

The specific capacitance (C_{sp}, F g^{-1}) of the electrode material can be calculated according to the following Equations (1) and (2):

$$C_{sp} = \frac{I\Delta t}{m\Delta V} \quad (1)$$

$$C_{sp} = \frac{\int IdV}{\upsilon m\Delta V} \quad (2)$$

where I is the discharge current (A), Δt is the discharge time (s), m is the mass of the active electrode material (g), ΔV is the discharge potential window (V) and ν is the scan rate (V s^{-1}).

The specific energy (E in Wh kg^{-1}) and specific power densities delivered (P in kW kg^{-1}) by an adopted symmetric supercapacitor based on hybrid materials were calculated using Equations (3) and (4), respectively:

$$E = \frac{C_{sp}V^2}{8 \times 3.6} \quad (3)$$

$$P = \frac{E}{t} \quad (4)$$

where C_{sp} is the specific capacitance of the electrode (F g^{-1}), V is the voltage (V) and t is the discharge time (s).

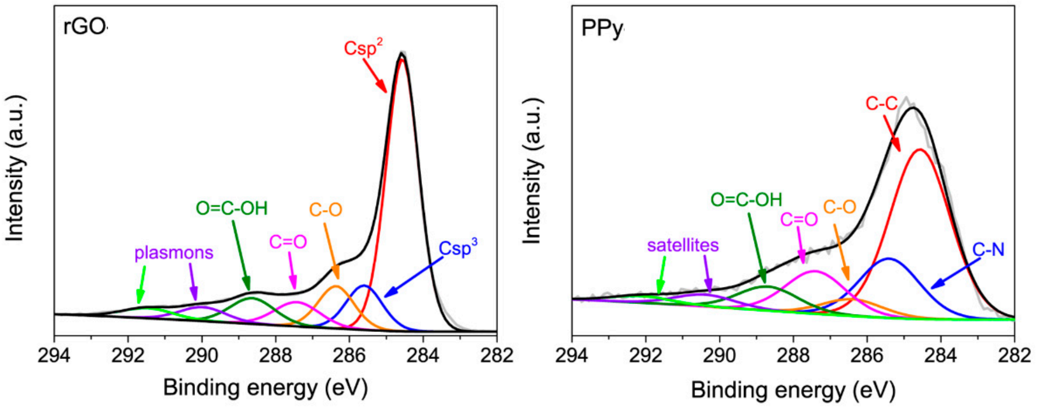

Figure S1. C1s high-resolution spectra of reduced graphene oxide (rGO) and polypyrrole (PPy).

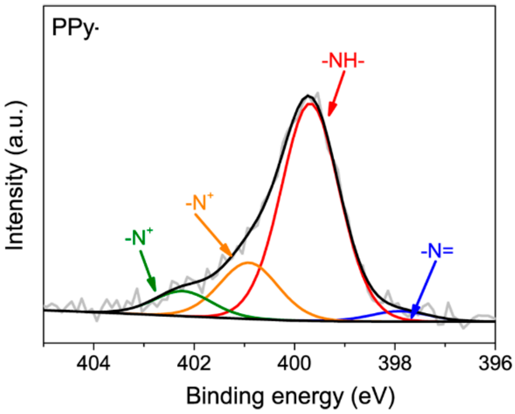

Figure S2. N1s high-resolution spectra of PPy.

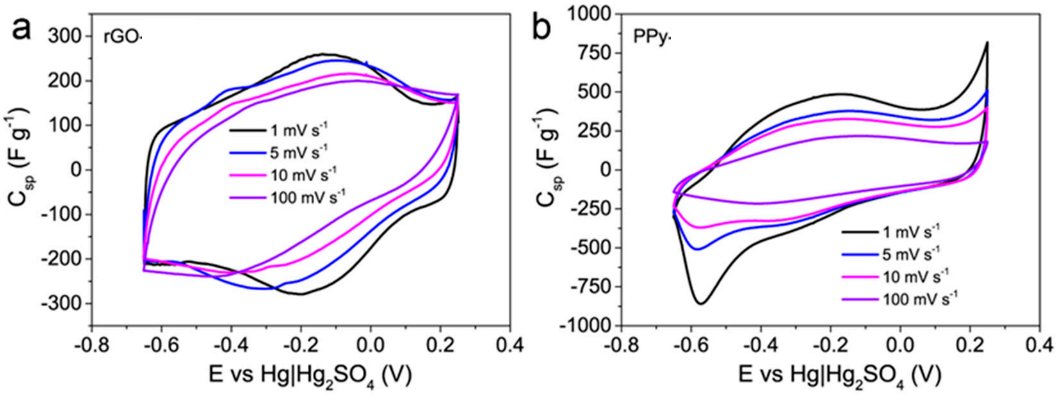

Figure S3. Cyclic voltammetry (CV) curves for (**a**) rGO and (**b**) PPy at the various scan rates in a 1 M H_2SO_4 electrolyte.

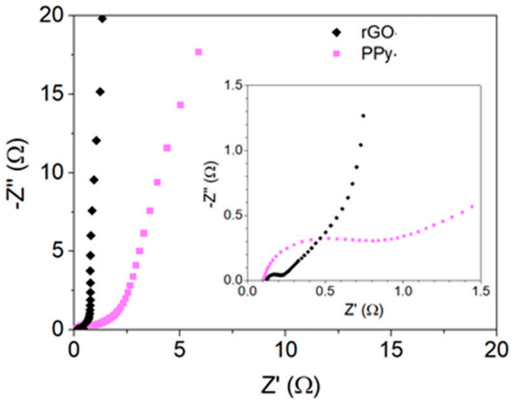

Figure S4. Nyquist plots of rGO and PPy with an inset representing the high-frequency range.

Table S1. Comparison of the PPy and carbon nanostructure composites' electrochemical performance in aqueous electrolytes.

Material	Specific Capacitance (F g^{-1})	Setup	Electrolyte	Cyclic Stability	Energy Density (Wh kg^{-1})	Ref.
PPy/RGO-10	255.7 at 0.2 A g^{-1}<br>199.6 at 25.6 A g^{-1}	2-EL	3 M KCl	83% after 4000 cycles	7	[1]
RGO-PPyNTs	280 at 10 mV s^{-1}<br>160 at 100 mV s^{-1}	3-EL	1 M KCl	77.3% after 1000 cycles	39	[2]
NTBP_PPy_Chem	91 at 2 mV s^{-1}<br>14 at 100 mV s^{-1}	3-EL	1 M H_2SO_4	30% after 10,000 cycles	not reported	[3]
GPG@MT	351 at 0.5 A g^{-1}<br>211 at 10 A g^{-1}	2-EL	1 M H_2SO_4	90% after 5000 cycles	not reported	[4]
PPy/GNS	315 at 0.5 A g^{-1}<br>220 at 5 A g^{-1}	3-EL	1 M H_2SO_4	96% after 1000 cycles	10.5	[5]
PPy-rGO-2	131 at 100 mV s^{-1}	2-EL	1M CH_3COOK/PVA	71% after 500 cycles	not reported	[6]
PPy/GO-HT-1:1	262 at 0.2 A g^{-1}<br>188 at 20 A g^{-1}	3-EL	1 M H_2SO_4	79% after 3000 cycles	7.4	This work
PPy/GO-HT-1:9	250 at 0.2 A g^{-1}<br>198 at 20 A g^{-1}	3-EL	1 M H_2SO_4	92% after 3000 cycles	7	This work

Abbreviations: PPyNTs—polypyrrole nanotubes; NTBP—multiwalled carbon nanotubes buckypaper; GPG@MT—rGO/PPy/rGO microtubes; GNS—graphene nanosheets.

References

1. Zhu, J.; Xu, Y.; Wang, J.; Wang, J.; Bai, Y.; Du, X. Morphology controllable nano-sheet polypyrrole–graphene composites for high-rate supercapacitor. *Phys. Chem. Chem. Phys.* **2015**, *17*, 19885–19894
2. Devi, M.; Kumar, A. In-situ reduced graphene oxide nanosheets–polypyrrole nanotubes nanocomposites for supercapacitor applications. *Synth. Met.* **2016**, *222*, 318–329
3. Iurchenkova, A.A.; Fedorovskaya, E.O.; Asanov, I.P.; Arkhipov, V.E.; Popov, K.M.; Baskakova, K.I.; Okotrub, A. V. MWCNT buckypaper/polypyrrole nanocomposites for supercapasitor application. *Electrochim. Acta* **2020**, *335*, 135700
4. Yang, J.; Weng, W.; Liang, Y.; Zhang, Y.; Yang, L.; Luo, X.; Liu, Q.; Zhu, M. Heterogeneous graphene/polypyrrole multilayered microtube with enhanced capacitance. *Electrochim. Acta* **2019**, *304*, 378–385
5. Zhang, D.; Zhang, X.; Chen, Y.; Yu, P.; Wang, C.; Ma, Y. Enhanced capacitance and rate capability of graphene/polypyrrole composite as electrode material for supercapacitors. *J. Power Sources* **2011**, *196*, 5990–5996

6. Abdul Bashid, H.A.; Lim, H.N.; Kamaruzaman, S.; Abdul Rashid, S.; Yunus, R.; Huang, N.M.; Yin, C.Y.; Rahman, M.M.; Altarawneh, M.; Jiang, Z.T.; et al. Electrodeposition of Polypyrrole and Reduced Graphene Oxide onto Carbon Bundle Fibre as Electrode for Supercapacitor. *Nanoscale Res. Lett.* **2017**, *12*, 1–10

© 2020 by the authors. Submitted for possible open access publication under the terms and conditions of the Creative Commons Attribution (CC BY) license (http://creativecommons.org/licenses/by/4.0/).
